# Epitaxial growth of SiGe films by annealing Al–Ge alloyed pastes on Si substrate

**DOI:** 10.1038/s41598-022-19122-7

**Published:** 2022-09-12

**Authors:** Keisuke Fukuda, Satoru Miyamoto, Masahiro Nakahara, Shota Suzuki, Marwan Dhamrin, Kensaku Maeda, Kozo Fujiwara, Yukiharu Uraoka, Noritaka Usami

**Affiliations:** 1 Graduate School of Engineering, Nagoya University, Furo-cho, Chikusa-ku, Nagoya, Aichi 464-8603 Japan; 2 Toyo Aluminium K.K., 341-14 Ohtani, Hino-cho, Gamo-gun, Shiga, 529-1608 Japan; 3 Nara Institute of Science and Technology, 8916-5 Takayama, Ikoma, Nara 630-0192 Japan; 4 Osaka University, 2-1 Yamadaoka, Suita, Osaka 565-0871 Japan; 5 Institute for Materials Research, Tohoku University, 2-1-1 Katahira, Aoba-ku, Sendai, Miyagi 980-8577 Japan

**Keywords:** Solar cells, Semiconductors, Surfaces, interfaces and thin films, Characterization and analytical techniques

## Abstract

A simple, low-cost, and non-vacuum epitaxial growth method to realize large-area semiconductors on crystalline silicon will become the game-changer for various applications. For example, we can expect the disruptive effect on the cost of large-scale III–V multi-junction solar cells if we could replace the high-cost germanium substrate with silicon–germanium (SiGe) on Si. For SiGe epitaxial growth, we attempted to develop a process using original Al–Ge pastes for screen printing and subsequent annealing. We compare two pastes including Al–Ge alloyed pastes with compositional uniformity in each particle and Al–Ge mixed pastes. We revealed that Al–Ge alloyed paste could form flatter SiGe film with much less residual pastes, supported by in-situ observations. The uniform and sufficient dissolution of the alloyed paste is responsible for these and led to higher average Ge-composition by annealing at 500 °C. The composition in SiGe was vertically graded up to ~ 90% at the topmost surface. These results show that printing and firing of Al–Ge alloyed paste on Si is the desirable, simple, and high-speed process for epitaxial growth of SiGe, which could be potentially used as the lattice-matched virtual substrate with III–V semiconductors.

## Introduction

Crystalline silicon solar cells have been widespread in the photovoltaic market, whereas their conversion efficiencies are approaching the Shockley-Queisser limit^1^. To overcome the theoretical upper limit, multi-junction solar cells have been developed by combining III–V semiconductors with different bandgaps. This architecture provides the highest efficiency in solar cells^2^ and has been commercialized mainly for space use. In early studies, a conversion efficiency has reached 40.7% by concentrator InGaP/InGaAs/Ge cells^3^ and 37.9% by triple-junction InGaP/GaAs/InGaAs cells^4^. Also, six-junction III–V solar cells reached conversion efficiencies of 39.2% for 1 sun and 47.1% for 143 suns^5^.

However, substrate materials used as bottom cells, such as Ge or GaAs, are quite expensive and difficult to achieve in large areas from an industrial point of view. Si substrate is desirable for implementing large-scale multi-junction solar cells because of its low manufacturing cost and high crystallinity^6^. Therefore, III–V compound solar cells on Si substrates have sustained interest for more than two decades^7^. Currently, high solar conversion efficiencies have been demonstrated on Si, fabricated by wafer bonding^8,9^ or mechanical stacking^10,11^, such as 32.6%^12^, 33%^13^, and 35.9%^14^ by 1 sun triple-junction of GaInP/GaAs/Si cells.

Meanwhile, a large lattice-mismatch exists between the Si substrate and the III–V semiconductors. This results in threading dislocations, which reduce the minority carrier lifetime and lower the open-circuit voltage of the cell^15,16^. For lattice-matching with each cell, silicon–germanium (SiGe) film on Si substrate has attracted attention in the controllability of a lattice constant and bandgap owing to the solid solution at all relative concentrations. Lattice-matching with upper cells and narrow gap can be realized by increasing Ge content higher than 82%^17^. Moreover, SiGe is low-cost and environmentally friendly and can be fabricated by chemical vapor deposition (CVD) or molecular beam epitaxy (MBE)^18,19^. However, they need toxic gasses such as SiH_4_ and GeH_4_^20–22^ or ultra-high vacuum and take much time. In general, lattice-mismatch still exists between Si and SiGe, and SiGe graded buffer layer is often used under SiGe bottom cell to reduce dislocation density^23,24^. In previous research, SiGe bottom cell fabricated on SiGe buffer layer, which was developed by AmberWave^25^, provided a low-dislocation interface for the nucleation of the lattice-matched III–V epitaxial layers^26^. This lattice-matched tandem structure reached a conversion efficiency of 20.6% by GaAsP/SiGe dual-junction solar cells grown on Si, with 20% P in GaAsP to match the lattice with ~ 82% Ge in SiGe^23^.

**Figure 1:**
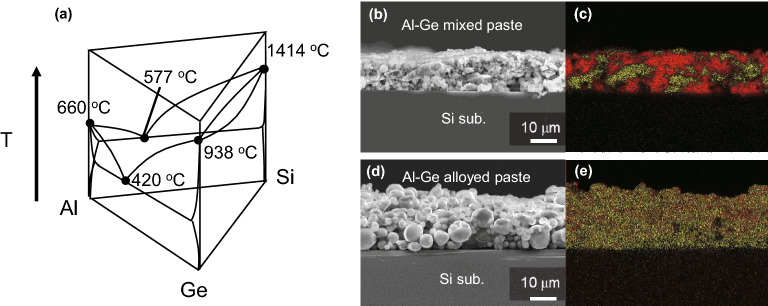
(**a**) Al–Si–Ge ternary phase diagram and cross-sectional SEM–EDX images of (**b**, **c**) Al–Ge mixed paste and (**d**, **e**) Al–Ge alloyed paste samples as-printed. Red and yellow color mappings by EDX indicate Al and Ge elements, respectively.

Recently, instead of conventional processes such as CVD and MBE, we have realized epitaxial growth of SiGe films having ~ 10 µm thickness by a process using original Al–Ge mixed pastes for screen printing on Si substrates and subsequent annealing in Ar ambient^27–29^. This method is a simple, low-cost, and high-speed approach that uses a melting point depression caused by the eutectic reaction, as seen in the Al-Si-Ge ternary phase diagram of Fig. 1a [see also ref.^30^]. Moreover, this process does not need toxic gases, and epitaxial growth can be realized in a non-vacuum atmosphere. However, the SiGe film grown on Si(001) has undulated interface derived from spearing reaction^28^ and non-uniform dissolution of Si substrate. The spearing reaction is induced by individual Al particles that reduce the native SiO_2_ on the Si substrate^31,32^. This leads to erosion of the Si surface in an inverted pyramid shape. In addition, a lot of Ge particles remain on the surface because of the insufficient dissolution of our conventional Al–Ge mixed pastes. These factors significantly affect device performances by leading to high defect density or obstacles in laminating the III–V cells and other processes such as surface polishing. To solve these problems, we developed Al–Ge alloyed pastes by atomizing Al and Ge particles, which have compositional uniformity in the individual particles. Figure 1b and e show cross-sectional scanning electron microscope (SEM) images combined with energy-dispersive X-ray spectroscopy (EDX) of as-printed samples by Al–Ge mixed paste and Al–Ge alloyed paste. In Fig. 1c and e, Al and Ge are mapped in red and yellow, respectively, representing elemental distributions in both pastes. In the mixed paste in Fig. 1b and c, Al and Ge particles are blended individually, and Al particles in contact with the Si substrate initiate spearing reactions. In contrast, in the alloyed paste in Fig. 1d and e, each particle is uniformly alloyed and there are no areas of contact between the substrate and individual Al particles. Therefore, the alloyed paste is expected to suppress spearing reactions and melt into Al–Ge liquid efficiently and uniformly at an eutectic point of ~ 420 °C (see Fig. 1a).

In this work, we compare SiGe films epitaxially grown by annealing Al–Ge alloyed pastes with Al–Ge mixed pastes screen-printed on Si, and demonstrate that the alloyed pastes enable to form relatively flat SiGe films with graded compositional structure and much less surface residue.

## Results

### Influence of annealing Al–Ge alloyed pastes on epitaxial growth of SiGe

**Figure 2:**
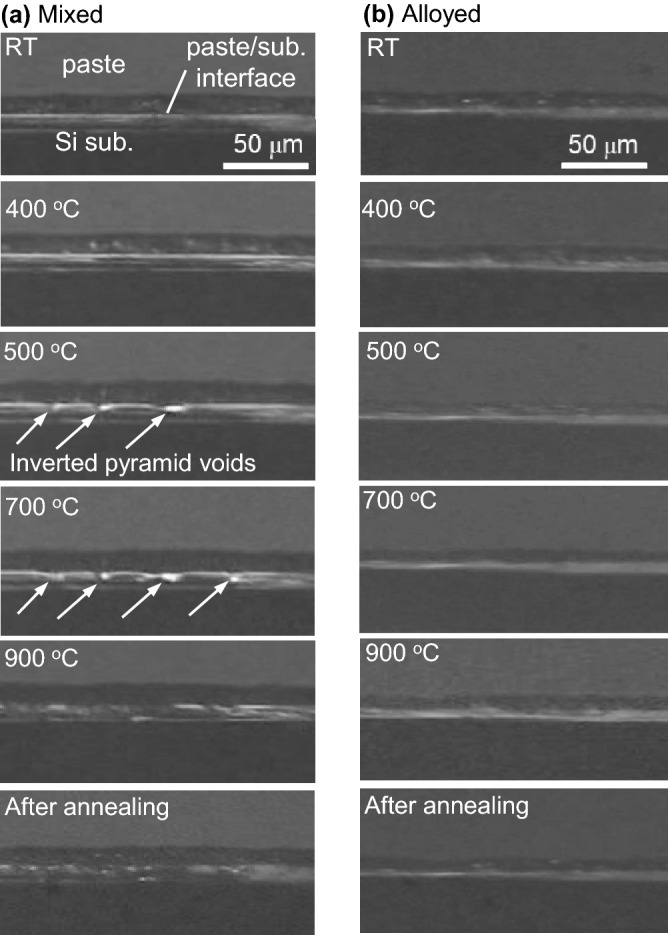
Cross-sectional optical images obtained during in-situ observation experiments of (**a**) mixed and (**b**) alloyed paste samples.

Figure 2 shows cross-sectional snapshots of samples taken at each temperature during the heat treatment process, which were obtained by *in-situ* observation of Al–Ge mixed and alloyed paste samples. These experiments were carried out to identify the Al-based eutectic growth process for each paste. It is seen that the substrate interface with Al–Ge mixed paste starts to be dissolved into the shape of an inverted pyramid indicated by arrows at around 500 °C, and the dissolution of the substrate is promoted with the voids growing as the temperature rises (Fig. 2a). These voids are attributed to the spearing reaction with Al particles and Si substrate. Then, the inverted pyramidal voids overlap at higher temperatures than 700 °C, eventually increasing the thickness of the Al-Si-Ge solution. After annealing, SiGe film with undulated substrate interface seems to be grown, while maintaining the substrate interface shape at the highest temperature of 900 °C. In contrast, no similar voids are observed for Al–Ge alloyed paste sample when the annealing temperature is elevated over 500 °C, as seen in Fig. 2b. At high temperatures above 700 °C, a relatively smooth substrate interface is observed since the alloyed paste is dissolved rather uniformly with eroding the whole of Si surface.. Therefore, the *in-situ* observation shows that the crystal growth proceeds with the dissolution of the substrate to define the SiGe/Si interface shape.

**Figure 3:**
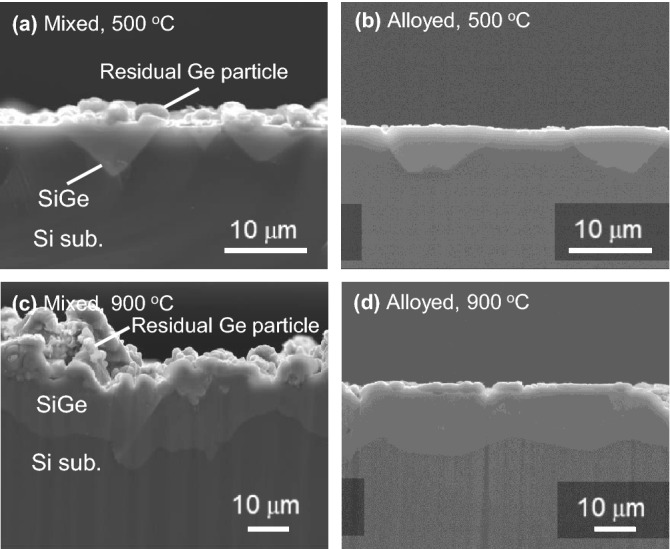
Cross-sectional SEM images of SiGe films fabricated by annealing two types of (**a**, **c**) mixed and (**b**, **d**) alloyed paste at 500 °C and 900 °C, respectively.

Figure 3 shows cross-sectional SEM images of samples grown by annealing Al–Ge mixed and alloyed pastes printed on Si. These samples were annealed at 500 °C and 900 °C, which are the onset temperature of the Si substrate dissolution and the top growth temperature used for the *in-situ* experiments, respectively. For the samples annealed at 500 °C, the SiGe films with an inverted pyramid shape were formed in the mixed paste sample by the spearing reaction as shown in Fig. 3a. On the other hand, a relatively flat bottom of the SiGe/Si interface was obtained for the alloyed paste sample as shown in Fig. 3b, which supports the suppression of the spearing reaction. Moreover, much less Ge residual pastes were found on the surface of SiGe film grown by the alloyed pastes, in contrast to the mixed pastes. At an annealing temperature of 900 °C, thicker and continuous SiGe film, which are also observed by *in-situ* observations, was confirmed with the partial formation of {111} facets near surfaces for both paste samples. The facet formation seems to be attributed to the minimization of the total energy. The decrease in the total surface energy by forming {111} facets could overcompensate the increase in the surface area and strain energy coming from the difference in the lattice constant between Si and Ge (∼4.2%), especially when the film becomes thicker and the Ge concentration increases^33^. The mixed paste sample has a SiGe film with undulated SiGe/Si interface and surface as shown in Fig. 3c. On the other hand, the alloyed paste allows forming a relatively flat SiGe film with much less Ge residue on the surface as shown in Fig. 3d, taking over the grooved interface shape formed in lower temperature annealing. The surface roughness of the samples was measured by laser microscopy. As a root means square (RMS) value for samples annealed at 500 °C, we obtained ~ 0.2 µm for the alloyed paste sample and ~ 0.9 μm for the mixed paste sample, respectively. For the alloyed paste sample, the reduction in Ge residues mainly contributes to the improved surface flatness at 500 °C, whereas there seems to be a small difference in the RMS values at 900 °C for both samples. The RMS of the alloyed paste sample at 900 °C becomes more significant to ~ 3.1 µm, which is much larger than that of a typical SiGe virtual substrate prepared by CVD and chemo-mechanical polishing (CMP). Importantly, the RMS value of the alloyed paste is much smaller than that of the mixed paste sample and the thickness of SiGe film, showing that the subsequent polishing process could be applied to obtain the smooth surface.

**Figure 4:**
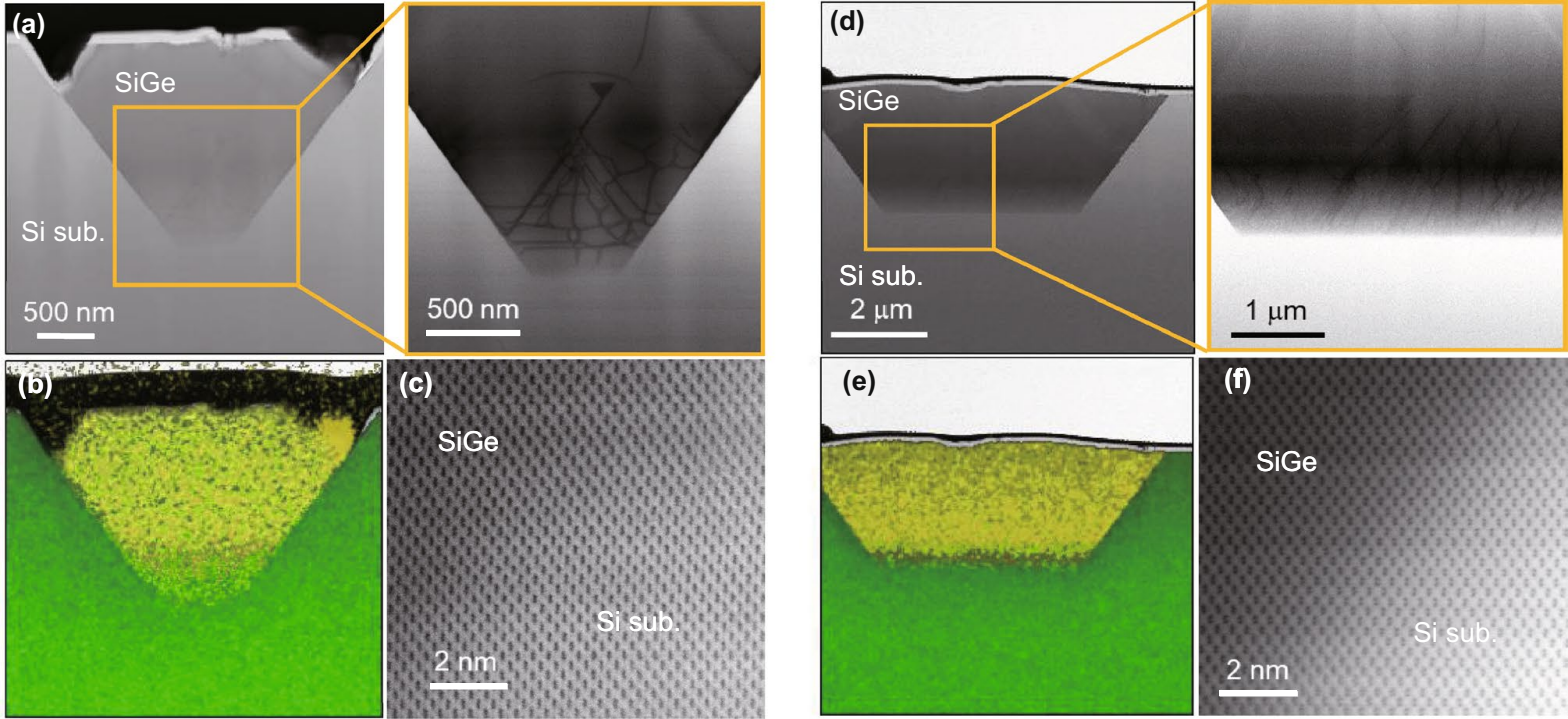
Cross-sectional STEM images of (**a**)–(**c**) mixed paste sample and (**d**)–(**f**) alloyed paste samples annealed at 500 °C. Lower panels of (**b**, **e**) show elemental mappings obtained by EDX, and yellow and green color mappings correspond to Ge and Si, respectively. (**c**, **f**) show lattice images by BF-STEM near SiGe/Si interfaces of the mixed and alloyed paste samples, respectively.

Bright-field scanning transmission electron microscopy (BF-STEM) images of SiGe annealed at 500 °C are shown in Fig. 4. Before observations, focused ion beam (FIB) processes were done, and samples were cut out to take the SiGe/Si bottom interface into the field of view in order to compare differences in the interfaces. It is seen that SiGe films grow with {111} facets, which has the lowest surface energy, in both mixed and alloyed paste samples at a low annealing temperature. STEM-EDX mappings also revealed that the alloyed paste forms SiGe with a flatter bottom of the SiGe/Si interface compared with mixed paste derived from the suppression of spearing reaction.

Dislocations are likely to concentrate at the bottom of the SiGe/Si interface in both samples since the strain energy tends to accumulate at the lowest regions of the inverted pyramids. The misfit dislocation density was calculated from the total length of the observed dislocations divided by the dimensions of the specimen processed by FIB. For both paste samples, the misfit dislocation density was estimated on the order of 10^8^–10^9^ cm^−2^. While the misfit dislocations predominantly occur at the substrate interface, the visible threading dislocations reaching the surface were hardly observed in the field of view. Therefore, the lowest reachable TDD can be reduced by increasing the thickness of the epitaxial layer. Furthermore, as can be seen in Fig. 4c and f, a part of crystal lattice at SiGe/Si interface is continuously connected in both paste samples. This indicates that SiGe grows epitaxially without any precipitations at the SiGe/Si interface.

### Characterization and analysis in compositional and strain distribution

**Figure 5:**
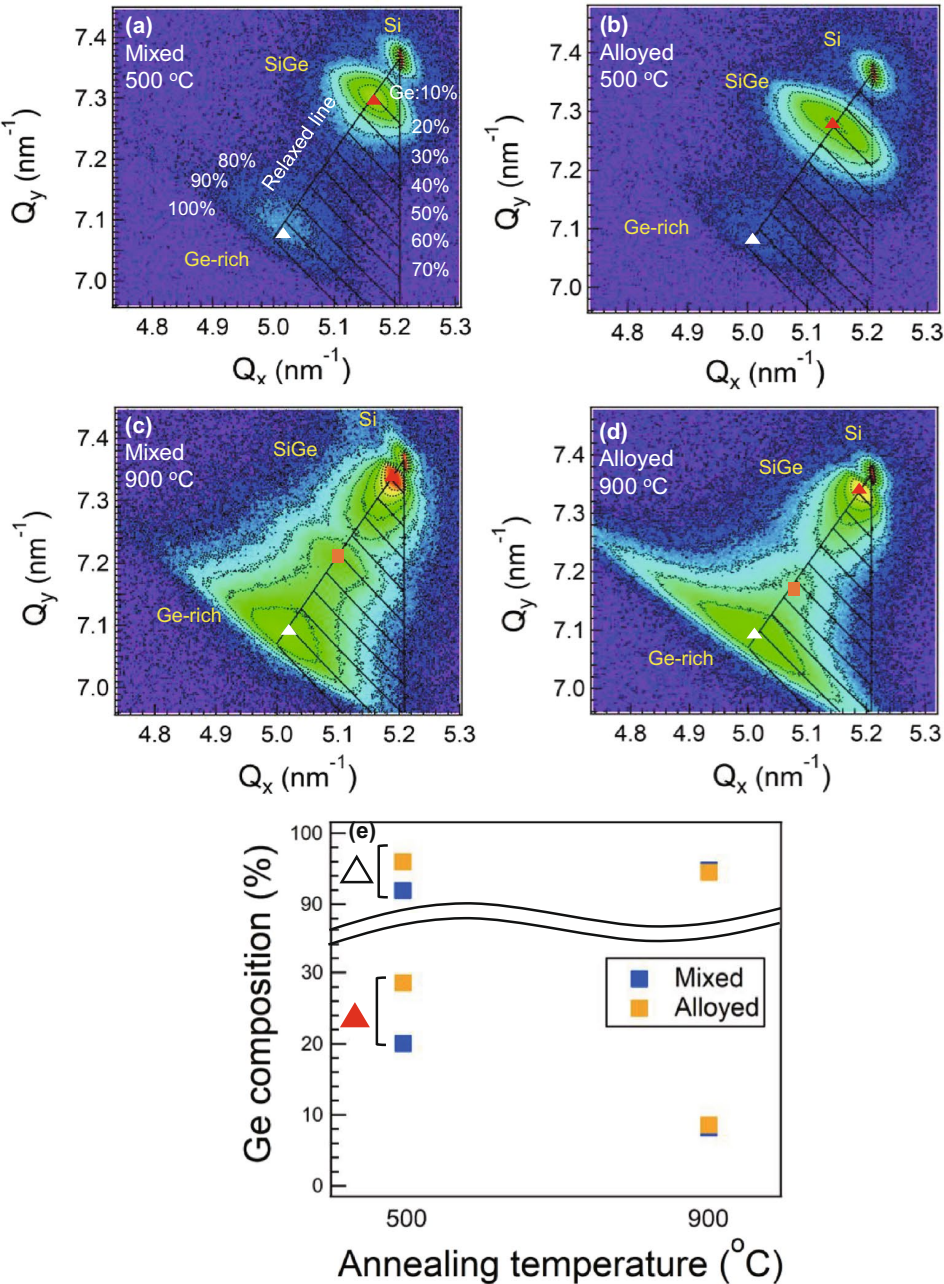
(**a**)–(**d**) (224) XRD-RSMs of SiGe epitaxial films grown on Si by annealing each paste at different temperature of 500 °C and 900 °C. (**e**) shows Ge composition by XRD-RSM derived from SiGe peaks of most intense peak () and Ge-rich peak (△), respectively.

Figure 5 displays (224) X-ray diffraction reciprocal space mappings (XRD-RSMs) of the SiGe films, where the upper and lower panels are obtained for the samples annealed at 500 °C and 900 °C, respectively. It is found that the diffraction peaks are distributed along fully relaxed lines connecting with Si and Ge reciprocal points, indicating that SiGe films are epitaxially grown on Si substrate. The most intense SiGe peaks () are observed at positions corresponding to a lower Ge-content of 8–30%. Besides, Ge-rich (~ 90%) peaks (△) also emerge in all samples. This indicates that the SiGe films are formed with high Ge content enough to lattice-match with III–V cells. The Ge-rich peak intensity increases at a high annealing temperature of 900 °C in Fig. 5c and d. At a low temperature, the Ge-rich peak intensity in the mixed paste is stronger than that in the alloyed paste due to a large amount of residual Ge paste left at the surface. Then, Si-rich peaks observed around ~ 20% are displaced towards the peaks of Si substrate as the annealing temperature increases, implying that SiGe intermixing proceeds at the film-substrate interface. Peaks with ~ 50% Ge () are also observed in both samples annealed at a high temperature of 900 °C, showing that several compositional distributions are achieved by the high-temperature annealing. The Ge composition extracted from each peak is compared in Fig. 5e. The most significant difference is that the Ge content related to the strongest SiGe peak () increases to around 30% for the alloyed paste sample annealed at a low temperature of 500 °C (Fig. 5e). Even for the Ge-rich peak (△), the Ge content is higher at 500 °C for the alloyed paste, although the peak intensity is smaller as seen in Fig. 5b. On the other hand, the difference in compositional distribution becomes smaller at 900 °C as shown in Fig. 5c and d, since the high-temperature annealing causes further dissolution of both pastes. It is seen that the alloyed paste sample has a broader and symmetric SiGe peak () across the fully relaxed line in the case of 500 °C annealing (Fig. 5b). The higher average Ge content results in the larger lattice mismatch with Si substrate. At the low temperature annealing, the increased strain energy would generate more misfit dislocations in the alloyed paste compared to the mixed paste, which enhances the mosaicity and strain distribution. Hence, the observed peak broadening in the alloyed paste sample can be attributed to the combined effects with mosaic spread, dislocation networks, and local residual strain.

**Figure 6:**
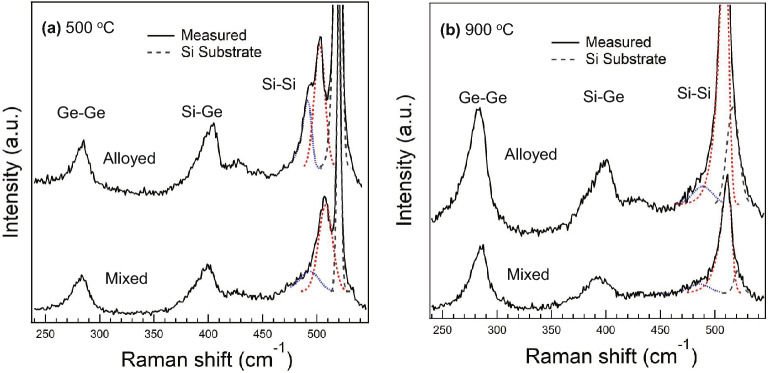
Raman spectra of SiGe films grown at (**a**) 500 °C and (**b**) 900 °C. The upper and lower spectra in each panel are obtained for the alloyed and mixed paste samples, respectively. Solid lines indicate experimental data. Each fitting component relevant to different Ge compositions are plotted by red dashed and blue dotted lines for clarity. Si–Si vibrational modes observed at around 420 cm^−1^ orginate from a local fluctuation of Ge atomic numbers in the vicinity of Si atoms.

**Figure 7:**
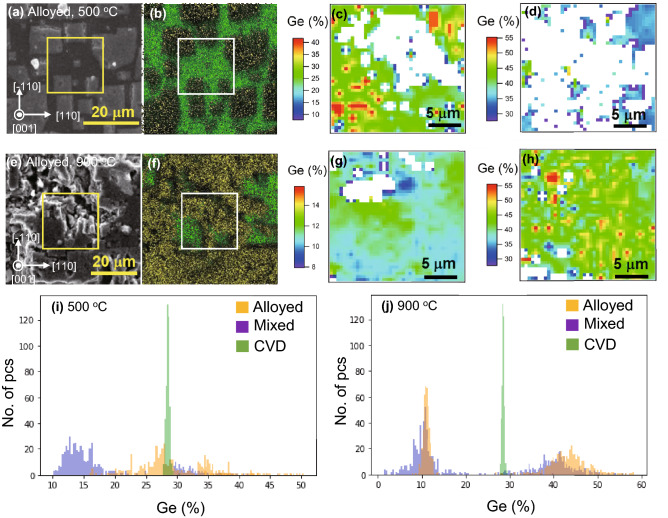
In-plain analysis of strain and composition for alloyed paste samples annealed at 500 °C and 900 °C. (**a**, **b**) and (**e**, **f**) show SEM–EDX images with color mappings of yellow and green indicating Ge and Si, respectively. (**c**, **d**, **g, h**) are mappings of two different distributions of Ge composition at areas marked with squares in SEM–EDX images, and calculated from (**c**, **g**) red dashed lines and (**d, h**) blue dotted lines of the Raman fitting curves shown in Fig. 6i, j show histogram of in-plane compositional distribution that are derived from Si–Si mode frequency in Raman spectra for SiGe films grown at 500 °C and 900 °C, respectively, comparing with SiGe grown by CVD indicated by green histogram. SiGe grown by annealing Al–Ge alloyed and mixed pastes are indicated by orange and purple, respectively.

Furthermore, Raman experiments provide spatial distributions of composition and strain near the SiGe surfaces. Typical Raman spectra were shown in Fig. 6, and in-plane compositional distributions were summarized in Fig. 7. The experimental data were obtained for 441 points in 20 μm × 20 μm area with 1 μm intervals on the sample surface. Figure 6a and b show the Raman spectra for both paste types of the samples annealed at 500 °C and 900 °C, respectively. Peaks in SiGe films have asymmetrical shapes with tails. For this reason, the fitting of each spectrum was performed by the exponentially modified Gaussian (EMG) function^34,35^. Compositions are extracted from the maximum positions of the Si–Si modes obtained by the fitting, which details are given in supplementary information.

For the spectra of 500 °C samples in Fig. 6a, the peaks related to the SiGe films are observed in three vibrational modes of Si–Si, Si–Ge and Ge–Ge. It is obvious that Si–Si mode, which is most sensitive to compositional fluctuation, contains two peaks from the SiGe layer with different Ge contents as indicated by the red dashed and blue dotted lines, respectively. In contrast, the Si–Ge and Ge–Ge modes are well fitted by a single EMG function. This could be explained by a small magnitude of compositional shift coefficients for these modes, and the impact of the different Ge content was effectively treated in a parameter to express the width of the peak [see supplementary information]. For the samples annealed at 900 °C in Fig. 6b, it is seen that each vibrational mode is expressed as a combination of multiple EMG functions in most of the measured area. This also indicates that high temperature annealing allows to introduce compositionally graded SiGe layers, which is in agreement with the XRD-RSM results.

The measurement areas were observed by SEM with EDX mappings to see the correspondence in each characterization. Figure 7a, b and e, f show surface SEM–EDX images of the alloyed paste samples annealed at 500 °C and 900 °C, respectively. Real-space mappings of Ge composition, which are calculated from each Si–Si mode frequency in Raman spectra, are shown in Fig. 7c, d and g, h. For the SiGe film grown at each temperature of 500 °C and 900 °C, the calculations of in-plane composition are carried out employing the Raman peaks indicated by red dashed and blue dotted lines observed in Si–Si mode (see Fig. 6a,b). A reasonable agreement with the SEM–EDX mappings obtained at the same locations ensures the validity of the in-plane Raman analysis. As can be seen, the alloyed paste samples show little Ge residue on the surface, and the grown SiGe regions can be clearly observed. In 500 °C annealing, surface SEM–EDX images show partial formation of the SiGe regions elongated in the <110> directions (see Fig. 7a,b). Then no Raman peaks derived from SiGe are observed in the area where SiGe films are not formed, and these non-calculated points are mapped in white color. Regarding the higher Ge-content area derived from the blue dotted peak, it is clear that many of the regions do not form SiGe with 30–40% Ge. As for 900 °C annealing in Fig. 7g and h, SiGe layer with ~ 50% Ge-content is formed more uniformly than at 500 °C in addition to the SiGe layer having ~ 10% Ge-content, which makes up a substantial portion of whole SiGe film. Considering that the SiGe films are compositionally graded in the growth direction, ~ 10% Ge-content layer can be covered by the topmost surface layer having a uniform ~ 50% Ge-content.

Figure 7i and j show histograms of calculated composition for each paste sample annealed at 500 °C and 900 °C, respectively. For comparison, the SiGe films grown by CVD with a target Ge composition of 30%, indicated by the green histogram, show compositional fluctuation in the range of ~ 3%. Comparing the histograms for each paste sample of 500 °C, the compositional distribution is higher in the alloyed paste sample, which can be correlated with the XRD results in Fig. 5a and b. In addition, Our SiGe grown by both paste forms two compositional ranges corresponding to red dashed and blue dotted peaks while the CVD sample has a single compositional range with small compositional distribution. For the samples annealed at 900 °C, ~ 50% Ge-content layers are revealed to form in larger areas in both paste samples compared to 500 °C annealing. The smaller distribution in the ~ 10% Ge-content layer for the alloyed paste implies that the ~ 10% Ge-content underlayer has in-plane compositional uniformity compared to the mixed paste. Additionally, the alloyed paste sample has a slightly higher compositional distribution in ~ 50% Ge-content layer, which approximately corresponds to the peak distribution in XRD-RSMs in Fig. 5d. On the other hand, the ~ 90% Ge-rich peaks measured by XRD in Fig. 5 cannot be observed in Si–Si mode due to the less number of Si atoms in the vicinity of other Si atoms, and are not observed in other modes in many measurement points of Raman spectroscopy. Therefore, based on the results of XRD and SEM, the Ge-rich layer containing ~ 90% Ge (△) is considered to be partially formed.

## Discussion

**Figure 8:**
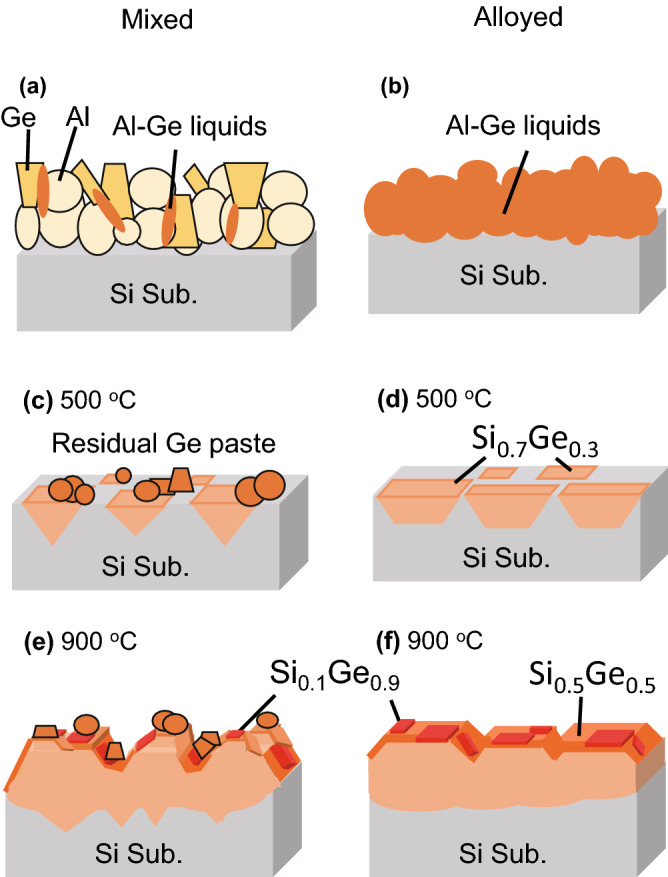
Schematic images of (**a**, **b**) paste dissolution process over Al–Ge eutectic point and (**c**)–(**f**) SiGe annealed at different temperature of 500 °C and 900 °C for mixed and alloyed paste samples, reflecting the results of structural characterizations such as concentration of dislocation at SiGe/Si interfaces and several layers which have different compositional distributions.

In Al–Ge mixed paste, Al–Ge liquid starts to form from interfaces between individual Al and Ge particles as shown in Fig. 8a. Meanwhile, in Al–Ge alloyed paste, Al and Ge are alloyed in each particle. Therefore, the Al–Ge eutectic reaction is promoted during the heat treatment, and the paste starts to melt simultaneously in each particle above the Al–Ge eutectic point of 420 °C (Fig. 8b). Owing to the efficient paste dissolution process, the alloyed paste is considered to suppress the spearing reaction caused by individually mixed Al particles. Then, the Al–Si–Ge ternary eutectic reaction proceeds evenly due to the uniform dissolution of the paste. These factors contributed to the improvement in film flatness and can lead to a reduction of defects at the SiGe/Si interface, which can improve device quality for future applications as virtual substrates. On the other hand, further improvement in the flatness of the substrate interface is still necessary by improving the processes. In addition, the alloyed paste dissolves sufficiently into Al–Ge liquid, which significantly reduces the amount of Ge residual paste on the surface. This is a great advantage because the Ge residual paste can be an obstacle in surface polishing by CMP in the future or stacking III–V upper cells.

As for the dislocations, SiGe fabricated by CVD is reported to have the TDD of 3 × 10^5^ cm^−2^ using SiGe buffer technology^26^, while the misfit dislocation density obtained in this work is on the order of 10^8^–10^9^ cm^−2^ and the TDD was hardly seen in the cross-sectional TEM. Further investigation based on defect etching reveals that the SiGe films grown in this work are found to have the TDD at most on the order of 10^7^–10^8^ cm^−2^ [see supplementary information]. Hence, our simple, low-cost, and high-speed process could realize SiGe films having a comparable quality to that grown by CVD.

As a summary of epitaxial growth of SiGe films, schematic images of two types of the paste samples annealed at different temperatures are shown in Fig. 8. These images highlight the SiGe film reflecting the results of structural characterization including compositional analyses. The difference in the paste type can be concluded to have a significant influence on the epitaxial growth manner of the SiGe films. In low-temperature annealing shown in Fig. 8c and d, Al–Si–Ge ternary eutectic reaction or SiGe growth starts, and dislocations concentrate at SiGe/Si interface. For Al–Ge mixed paste, SiGe film has grown in an inverted pyramidal shape originated from spearing reaction by Al particles, and insufficient dissolution of the paste left a lot of Ge residue on the surface. On the other hand, Al–Ge alloyed paste with compositional uniformity in each particle shows a flatter SiGe/Si (100) interface and remarkable reduction of Ge paste residue due to uniform and sufficient paste dissolution. Compositional characterizations provide distributions in reciprocal and real space. As mentioned before, the average Ge composition is increased by annealing the alloyed pastes at a low temperature. This is also contributed by sufficient formation of the Al–Ge liquid phase on Si substrate even at the low temperature above its binary eutectic point, which leads to producing more Ge liquid. Besides, the dissolution of Si substrate can be suppressed at around 500 °C. These facts enable the formation of SiGe film with a high average Ge content at a low temperature. In high-temperature annealing shown in Fig. 8e and f, epitaxial growth progressed while maintaining the shape of SiGe/Si interface at the beginning of the substrate dissolution, and thicker and continuous SiGe films have grown with {111} facets near the surfaces. In addition, high Ge-content layers with ~ 50% and ~ 90% Ge are formed toward the surface revealed by Raman spectra, resulting in vertically graded compositional structures. XRD-RSMs in Fig. 5 and SEM–EDX line analysis results in supplementary information also support the result of graded structure. The compositional grading in SiGe film is preferable for the bottom cell application in terms of defect suppression, and it is indicated that a favorable compositional distribution is formed by high-temperature annealing. More gradual vertical compositional distribution and suppression of misfit dislocations might be achieved by optimizing heating and cooling rates in the annealing procedures. Moreover, mixed acid solution at a ratio of H_3_PO_4_: CH_3_COOH: HNO_3_: H_2_O = 16:1:1:2 is often used to etch Al–Ge residual pastes. Since this etching solution removes Ge-rich layer with ~ 90% Ge, the Ge-rich peak cannot be previously detected by XRD [see supplementary information]. In this work, diluted HF etching was performed with improved selectivity for Al enabling us to leave the Ge-rich layer on the surface, which can lattice-match with III–V semiconductors. The Ge-rich layer is considered to be partially grown, and indeed the Ge-rich peak in the alloyed paste sample becomes more intense and broader in XRD-RSMs, as shown in Fig. 5d. For the alloyed paste sample, efficient formation of Al–Ge liquid results in growing a thicker Ge-rich layer in a larger area. Due to the increase in strain energy, the number of dislocations in the Ge-rich layer would be higher in the alloyed paste than in the mixed paste. As for the surface of the SiGe films, the undulated surface is formed in the mixed paste because of non-uniform Al-Si-Ge ternary eutectic reaction, whereas flatter surface and interface are formed in the alloyed paste sample owing to the more uniform ternary reaction. The surface roughness still has room for improvement, but the roughness is smaller than the thickness of SiGe film in alloyed pastes. Therefore, it should be possible to obtain SiGe films with a flat surface by polishing the surfaces of the Al–Ge alloyed paste sample. The planalized surface can be also helpful for preventing dislocation pile-ups in subsequent layers, according to an original effort on the CVD-grown SiGe films^36^. In that way, we have obtained SiGe films by the simple high-speed process of screen printing and annealing Al–Ge alloyed paste, which could act as the lattice-matched virtual substrate with III–V semiconductors after improving several issues. Especially, it is essential to improve film flatness further by surface polishing such as CMP, lower the number of dislocations by controlling to more gradual composition distribution, and form the uniform Ge-rich (~ 90% Ge) layer to realize lattice matching with upper III–V cells and high conversion efficiency.

## Conclusion

SiGe films were fabricated on Si(100) using Al–Ge alloyed paste with compositional uniformity in the paste to improve the issues of film flatness and Ge residual paste. Compared with conventional Al–Ge mixed paste sample including *in-situ* observation of Al-based eutectic epitaxial growth process, we found that annealing of Al–Ge alloyed pastes allows to form SiGe films with relative flatness and much less Ge residue on the surface thanks to the uniform and sufficient dissolution of the paste. The improvements in flatness in SiGe/Si (100) interface and surface Ge residue can be a significant contribution to further process improvement in the future. Moreover, 8.5% higher average Ge content was achieved in low-temperature annealing. Other structural analyses revealed that high-temperature annealing enables vertically graded SiGe films with partial Ge-rich (~ 90% Ge) layers on the surfaces. Consequently, the annealing of Al–Ge alloyed paste is a promising method that can grow SiGe films with a low-cost, simple, and high-speed process. Further improvements in film flatness, dislocation, and uniform formation of Ge-rich layer are essential for bottom cell applications.

## Methods

### Fabrication process of SiGe epitaxial film

Two different types of pastes were used; an individually mixed paste with Al and Ge particles, and an alloyed paste prepared by atomizing process of both particles. As for the particle size, Al particles in the mixed paste are ~ 7 µm and Ge particles are in angular shape with different particle sizes less than 44 µm by 325 mesh passing. The alloyed pastes have similar particle size distribution as Al particles in the mixed paste. Each paste has a mole fraction of Al: Ge = 7:3, which is the eutectic composition of Al and Ge, and the paste thickness is 20 µm, respectively. Then, these pastes were screen-printed on Si(100) substrates with sizes of 2 cm × 2 cm and dried at 100 °C for 10 min by Toyo Aluminium. Subsequently, SiGe epitaxial films were grown by rapid thermal annealing for 5 min at different temperatures with a heating rate of about 3 °C/s. Here furnace atmosphere was replaced by argon gas to prevent the surface from oxidation. After annealing, the furnace was quenched. Al residues derived from the pastes on the surface were selectively removed by diluted HF (HF: H_2_O = 1:10) etching with immersing samples for ~ 15 h at room temperature. In addition, the surfaces of samples were softly polished with cotton swabs after the etching for further removal of the residue.

### Material characterization

Film appearances were compared between the mixed and alloyed samples by SEM combined with EDX and BF-STEM. States of dislocations were also observed by BF-STEM. Furthermore, the SiGe epitaxial growth during the annealing process was closely investigated by an in-situ observation furnace system, where digital microscope images were continuously recorded in synchronization with temperature logging. The reciprocal scape composition and strain of SiGe films were characterized by (224) XRD-RSMs with Cu *Kα* radiation. The in-plane composition/strain distributions were analyzed by micro-Raman scattering spectroscopy, where laser wavelength and power of the excited laser were set at 488 nm and ~ 4 mW, respectively. The penetration depth of the laser depends on the Ge composition, and is estimated as ~ 60 nm when the Ge composition is ~ 50%. The in-plane compositions and strains were calculated from the peak positions of Ge–Ge, Si-Ge, and Si–Si vibration modes. In calculation, composition and strain-shift parameters^37,38^ were used.

## Supplementary Information

Supplementary Information.

## Data Availability

The datasets used and/or analysed during the current study available from the corresponding author on reasonable request.
