# Supplementary material for: Epitaxial growth of SiGe films by annealing Al–Ge alloyed pastes on Si substrate
Source: Sci Rep. 2022 Sep 12;12:14770. doi: 10.1038/s41598-022-19122-7 (PMC9467981; doi:10.1038/s41598-022-19122-7)
Supplement: Supplementary file 1 — Supplementary Information. [file 41598_2022_19122_MOESM1_ESM.pdf]

Epitaxial growth of SiGe films by annealing Al-Ge alloyed pastes on Si substrate

Keisuke Fukuda[1*], Satoru Miyamoto[1], Masahiro Nakahara[1,2], Shota Suzuki[2,3,5], Marwan Dhamrin[2,3], Kensaku Maeda[4], Kozo Fujiwara[4], Yukiharu Uraoka[5], and Noritaka Usami[1**]

[1] Graduate School of Engineering, Nagoya University, Furo-cho, Chikusa-ku, Nagoya 464-8603, Japan

[2] Toyo Aluminium K.K., 341-14 Ohtani, Hino-cho, Gamo-gun, Shiga 529-1608, Japan

[3] Osaka University, 2-1 Yamadaoka, Suita, Osaka 565-0871, Japan

[4] Institute for Materials Research, Tohoku University, 2-1-1 Katahira, Aoba-ku, Sendai 980-8577, Japan

[5] Nara Institute of Science and Technology, 8916-5 Takayama, Ikoma, Nara 630-0192, Japan

*Correspondence to fukudak0801@gmail.com

**usa@material.nagoya-u.ac.jp

Supplementary Information

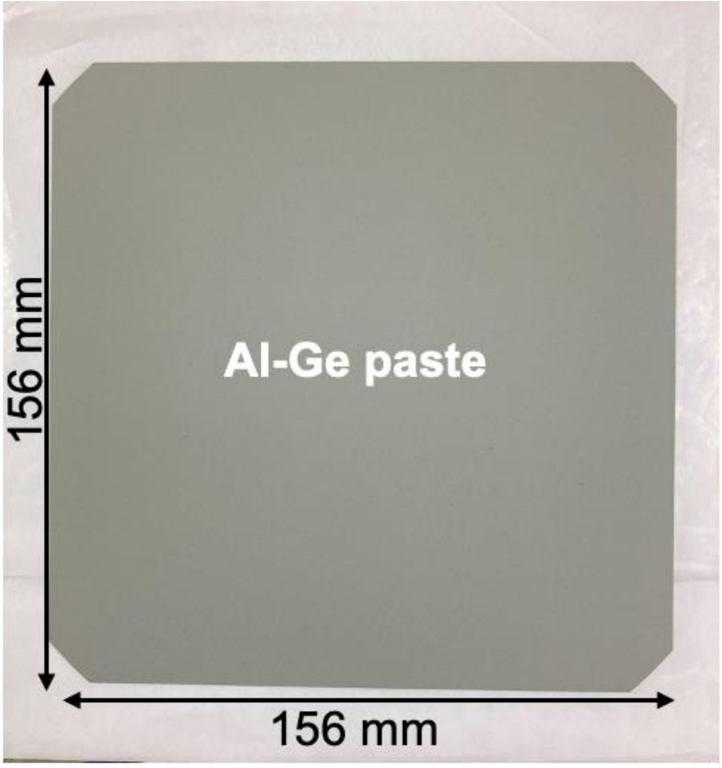

Figure S1. Photograph of screen-printed Al-Ge paste on a 156 mm square Si substrate. As shown in Fig. S1, it is possible to print Al-Ge pastes in the size of the standard solar cells, and to grow SiGe films on a large area by annealing in belt furnaces. In Article, the largely screen-printed samples were cleaved to smaller sizes and annealed under different conditions.

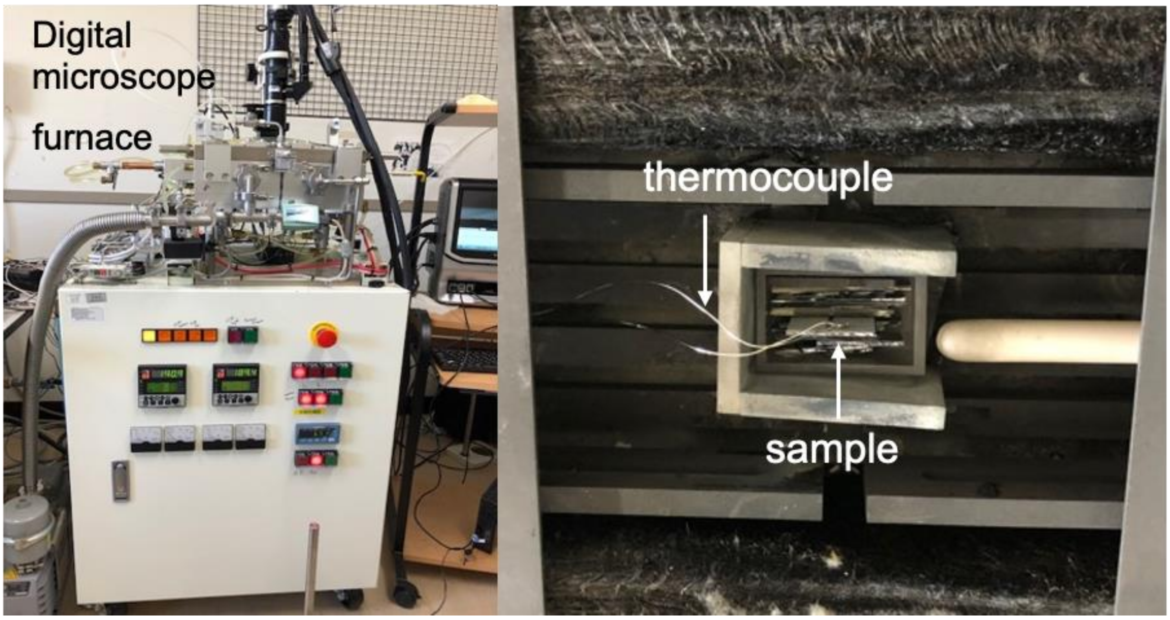

Figure S2. Photographs of a special furnace for the *in-situ* observation and a sample in a quartz crucible in the furnace. The sample was placed vertically between Si dummy wafers in the crucible and put into the furnace. Samples were heated up to a target temperature at a rate of approximately 40 °C/min. The cross-section of the samples was observed by the digital microscope during the annealing process, and images were continuously recorded. The annealing temperature was calibrated by a thermocouple on the sample surface.

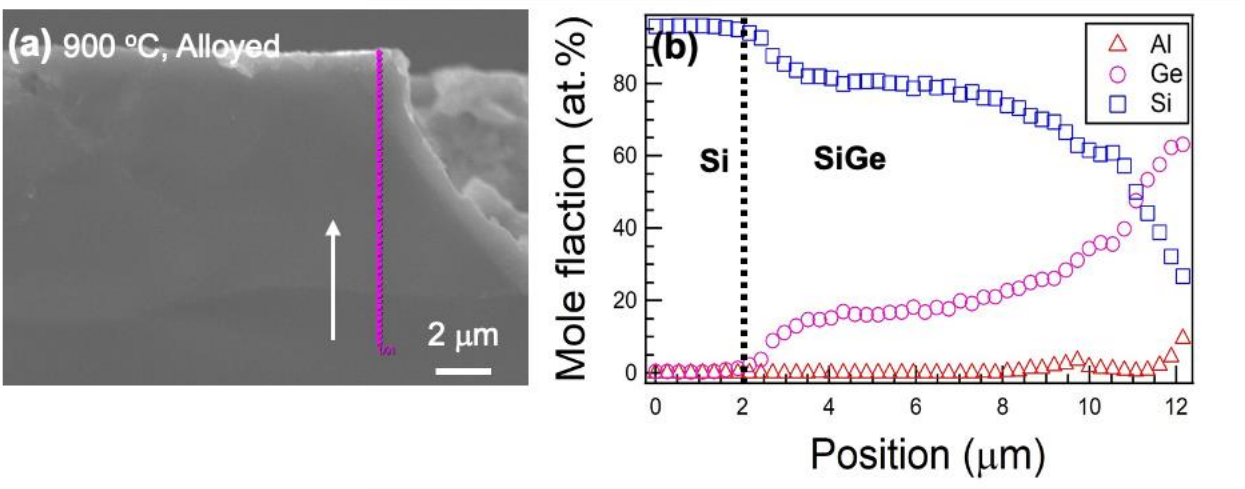

Figure S3. Cross-sectional analysis by (a) SEM and (b) EDX. SiGe film annealed at 900 °C has vertically graded compositional distributions towards the surfaces, with relatively steep gradients at the SiGe/Si interface and near the surface.

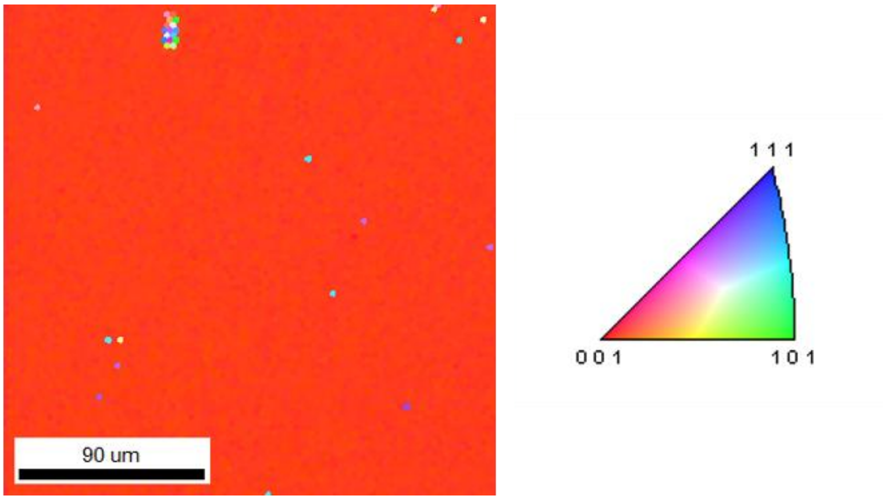

Figure S4. Electron backScatter diffraction (EBSD) image of SiGe grown by annealing Al-Ge alloyed paste on Si(100) at 500 °C to show that SiGe is monocrystalline and epitaxially grown on Si(100). The several spots with different orientations come from the Ge residue.

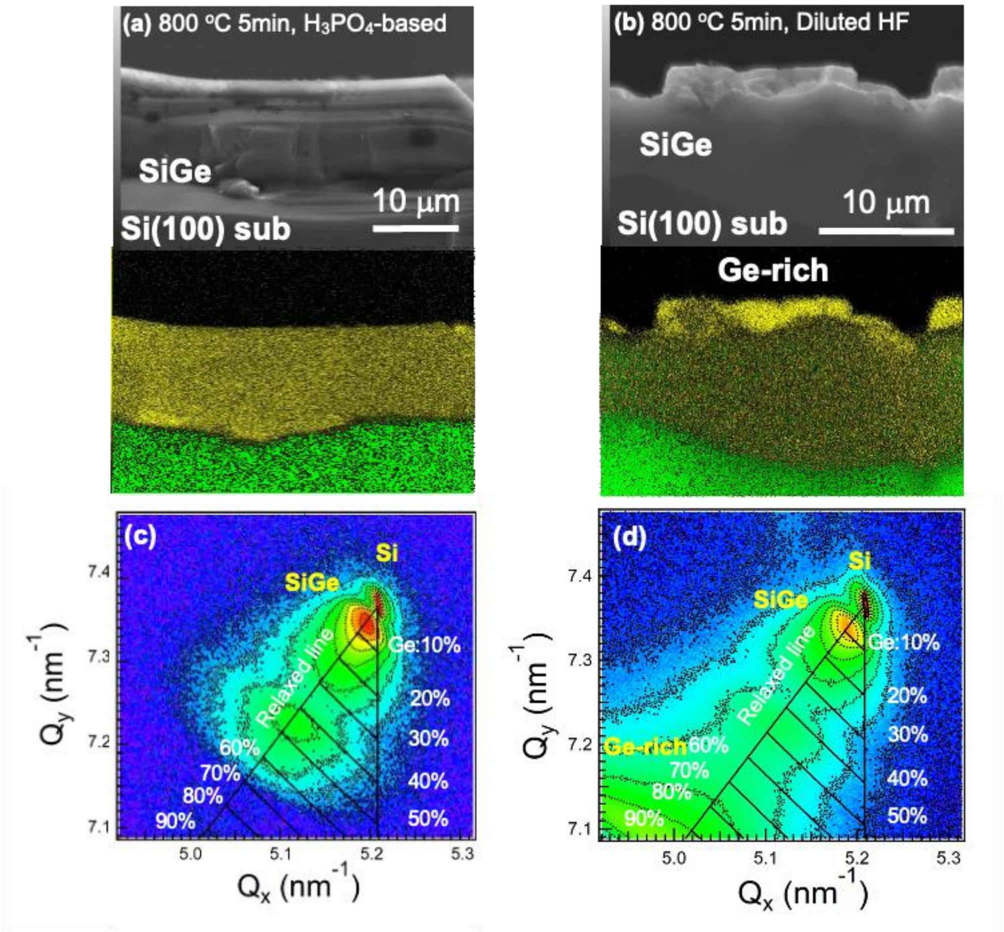

Figure S5. Improvement in etching technique for removal of residual Al paste. (a)(b) and (c)(d) show SEM-EDX images and (224) XRD-RSMs of Al-Ge mixed paste samples annealed at 800 °C, respectively. Color mappings by yellow (●) and green (●) indicate Ge and Si, respectively. In the conventional method in Figs. S5(a) and S5(c), H_3PO_4-based etching solution has been used, and the solution consists in the ratio of H_3PO_4: CH_3COOH: HNO_3: H_2O = 16:1:1:2. However, the etching with H_3PO_4-based solution removes the Ge-rich (~90% Ge) layer on the surface in addition to Al residue, and its peak disappears in RSM. On the other hand, diluted HF (HF: H_2O = 1:10) has good selectivity for Al, and we could remove Al residual paste while keeping the Ge-rich layer on the surface after immersion for 15 hours as confirmed in Fig. S5(b) and S5(d). The time to etch the Al residue could be shortened by etching conditions.

SI Section S1 - Fitting of Raman spectra in SiGe

In SiGe grown by our method, peaks in Raman spectra have asymmetric shapes due to compositional gradation or strain in SiGe. Therefore, to take this asymmetry into account, the following exponentially modified Gaussian (EMG) function[1,2] line shape is used,

$$I_{\mathrm{EMG}}(\omega) = \frac{a}{2s}\exp\left(\frac{w^2}{2s^2} + \frac{\omega_0-\omega}{s}\right) \times \left[\operatorname{erf}\left(\frac{\omega_0-\omega}{\sqrt{2}w} - \frac{w}{\sqrt{2}s}\right) + \frac{s}{|s|}\right], \quad (1)$$

where a is a peak area, ω_0 is the mode frequency (unless the peak is symmetric, ω_0 does not match the maximum value of the EMG function, and therefore we use maximum peak position of the function (ω_{max}) for calculation), w is the width, and s is the asymmetry parameter. This asymmetry parameter is negative when the peak is broadened asymmetrically to lower frequency.

SI Section S2 – Calculation of composition and strain from Raman spectra

The peak position of each vibration mode in Raman spectrum shifts due to composition and strain in SiGe. From the maximum peak positions ω_{max} of Si-Si, Si-Ge and Ge-Ge modes obtained by EMG fittings, we can calculate Ge composition $\boldsymbol{x}$ using the following bilinear function of $\boldsymbol{x}$ and in-plane strain $\boldsymbol{\varepsilon}_{\|}$, as given by

$$\boldsymbol{\omega^{SiSi} = \omega_0^{SiSi} + A^{SiSi}x + b^{SiSi}\varepsilon_{\|}} \quad (2)$$
$$\boldsymbol{\omega^{SiGe} = \omega_0^{SiGe} + A^{SiGe}x + b^{SiGe}\varepsilon_{\|}} \quad (3)$$
$$\boldsymbol{\omega^{GeGe} = \omega_0^{GeGe} + A^{GeGe}x + b^{GeGe}\varepsilon_{\|}} \quad (4)$$

where $\boldsymbol{\omega^{SiSi}}$, $\boldsymbol{\omega^{SiGe}}$ and $\boldsymbol{\omega^{GeGe}}$ are the maximum peak positions obtained by Raman, and $\boldsymbol{\omega_0^{SiSi}}$= 520 cm^{-1}, $\boldsymbol{\omega_0^{SiGe}}$= 400.5 cm^{-1} and $\boldsymbol{\omega_0^{GeGe}}$= 280 cm^{-1} are used here. $\boldsymbol{A^{SiSi}}$(= -75 cm^{-1}), $\boldsymbol{A^{SiGe}}$(= 16 cm^{-1}) and $\boldsymbol{A^{GeGe}}$(= 19 cm^{-1}) are linear coefficients with Ge composition $\boldsymbol{x}$, on the other hand, $\boldsymbol{b^{SiSi}}$(= -870 cm^{-1}), $\boldsymbol{b^{SiGe}}$(= -575 cm^{-1}), $\boldsymbol{b^{GeGe}}$(= -510 cm^{-1}) are strain-shift coefficients[3,4] with $\boldsymbol{\varepsilon}_{\|}$. Depending on differences in absolute values of the composition-shift coefficient, some peaks of Ge-Ge and Si-Ge modes, which have smaller coefficients than Si-Si mode, are covered by other peaks and cannot be fitted by the above function. $\boldsymbol{\omega^{SiGe}}$ cannot be written as a linear function of $\boldsymbol{x}$ for the full range 0 < $\boldsymbol{x}$ < 1. In Alrticle, the composition was calculated by a simple linear equation using only Si-Si modes by assuming $\boldsymbol{\varepsilon}_{\|}$ = 0 to investigate in-plain non-uniformity of the sample, as given by

$$\boldsymbol{\omega^{SiSi} = \omega_0^{SiSi} + A^{SiSi}x}$$

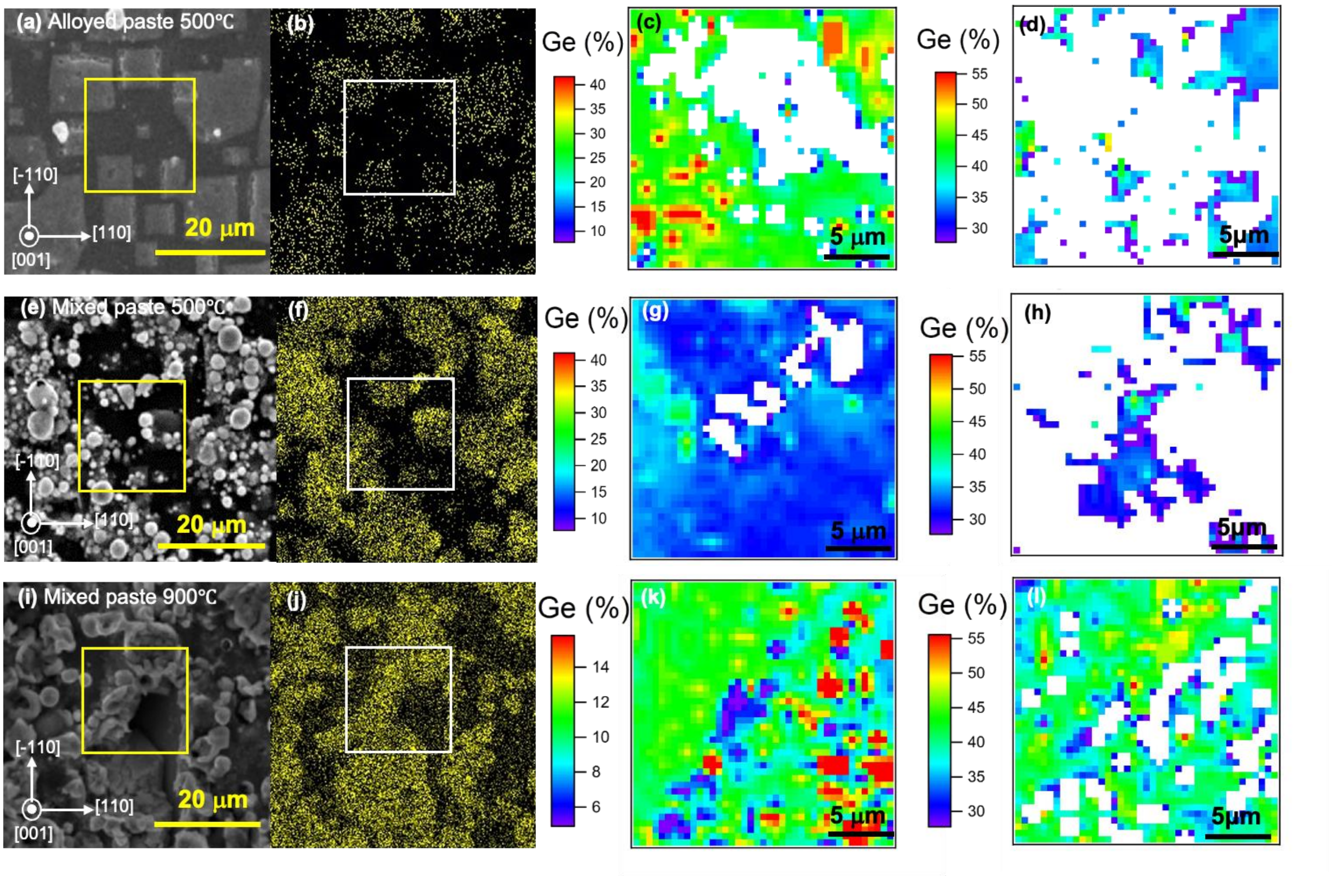

Figure S6. Surface SEM-EDX images and Raman real space mappings measured at the corresponding locations for alloyed paste sample annealed at 500 °C and conventional Al-Ge mixed paste samples at 500 °C and 900 °C. Yellow (●) and green (●) mappings in EDX indicate Ge and Si, respectively. In contrast to the alloyed paste in Figs. S6(a) and S6(b), the mixed paste samples in Figs. S6(e,f) and S6(i,j) show a large amount of Ge residue due to the insufficient dissolution of the paste. In Al-Ge mixed paste, the elemental mappings by each characterization of EDX and Raman agree well, indicating that quantification of in-plane composition was successfully done by Raman spectroscopy. In high-temperature annealing at 900 °C, the upper layer with ~50% Ge is identified on the underlayer with ~10% Ge more uniformly than 500 °C. The compositional distribution ranges agree well with that of XRD-RSMs.

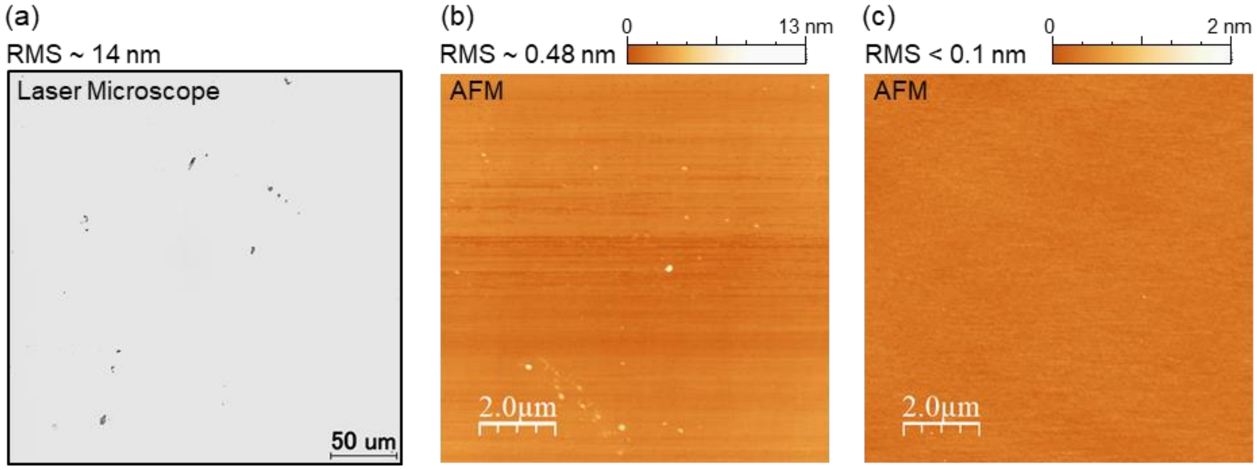

Figure S7. (a) Laser microscope image and (b) AFM image taken after the standard CMP process of the paste-grown SiGe sample having Ge-rich surface. (c) Comparison of the surface roughness with the polished $Si_{0.7}Ge_{0.3}$ samples grown in a conventional CVD manner. The surface roughness can be drastically improved by the standard CMP process. On the SiGe film obtained after the CMP, the RMS value is ~14 nm, which was determined for a relatively large area of 300-µm-length scale employing the laser microscope [see Figure S7(a)]. Obviously, this value is at least an order of magnitude lower than a typical value of 0.2-0.9 µm, similarly obtained for unpolished samples after selective etching of Al pastes. The RMS value of AFM analysis in Figure S7(b) was found to be ~0.48 nm for an area of 10 µm×10 µm. This RMS value is on the same level as ~0.1 nm which is normally obtained on the polished surface of the CVD-grown SiGe samples [compare with Supporting Figures S7(b) and S7(c)]. These facts demonstrate that the CMP process can be adopted to our paste-grown samples, in order to obtain a flat SiGe surface sufficient for the regrowth of upper III-V layers.

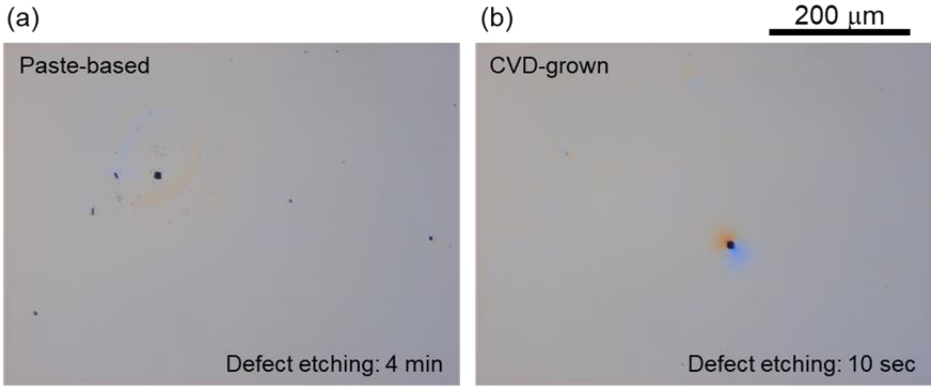

Figure S8. Differential interference microscope images of surface etch-pits obtained on planarized surface by defect etching based on Cr-free acid solutions of HF: HNO_3: CH_3COOH = 1:15:12; (a) Ge-rich SiGe sample grown by annealing Al-Ge alloyed pastes at 900 °C and (b) CVD-grown $Si_{0.7}Ge_{0.3}$ sample. Here the etching rate on each sample surface was determined from a step height at the boundary of partially protected area with wax. The etching rate was ensured to be ~1.7 µm/min for CVD-grown $Si_{0.7}Ge_{0.3}$ sample surface, in good agreement with the previous study reported on the defect etching of $Si_{1-x}Ge_x$ surface.[5] Similarly, the etching rate was determined to be around ~0.7 µm/min for our Ge-rich SiGe films grown by the Al-paste technique. These facts enable us to perform a precise control of etching depth for etch-pit observation employing differential interference microscope. In Figs S8(a) and S8(b), characteristic interference patterns reflecting etch-defined facets can be clearly seen in the proximity of the threading dislocations. Hence, the SiGe films grown in this work are found to have the etch-pit density 3-10 times larger than CVD-grown SiGe samples, suggesting that the threading dislocation density is at most on the order of 10^7-10^8 cm^{-2}.

References

1. Lockwood, D. J. & Wasilewski, Z. R. Optical phonons in $Al_xGa_{1-x}As$: Raman spectroscopy. *Phys. Rev. B - Condens. Matter Mater. Phys.* **70**, 155202 (2004).
2. D'Costa, V. R., Tolle, J., Poweleit, C. D., Kouvetakis, J. & Menéndez, J. Compositional dependence of Raman frequencies in ternary $Ge_{1-x-y}Si_xSn_y$ alloys. *Phys. Rev. B - Condens. Matter Mater. Phys.* **76**, 035211 (2007).
3. Aktas, O. *et al.* Laser-Driven Phase Segregation and Tailoring of Compositionally Graded Microstructures in Si-Ge Nanoscale Thin Films. *ACS Appl. Mater. Interfaces* **12**, 9457–9467 (2020).
4. Perova, T. S. *et al.* Composition and strain in thin $Si_{1-x}Ge_x$ virtual substrates measured by micro-Raman spectroscopy and x-ray diffraction. *J. Appl. Phys.* **109**, 033502 (2011).
5. Abbadie, A. *et al.* Defect delineation and characterization in SiGe, Ge and other semiconductor-on-insulator structures. *Solid-State Electron.* **53**, 850 (2009).
